# The effect of vernal solar UV radiation on serum 25-hydroxyvitamin D concentration depends on the baseline level: observations from a high latitude in Finland

**DOI:** 10.1080/22423982.2016.1272790

**Published:** 2017-01-20

**Authors:** Toni Karppinen, Meri Ala-Houhala, Lasse Ylianttila, Hannu Kautiainen, Kaisa Lakkala, Henna-Reetta Hannula, Esa Turunen, Heli Viljakainen, Timo Reunala, Erna Snellman

**Affiliations:** ^a^ Medical School, University of Tampere, Tampere, Finland; ^b^ Department of Dermatology, Tampere University Hospital, Tampere, Finland; ^c^ Department of Dermatology, Päijät-Häme Central Hospital, Lahti, Finland; ^d^ Non ionizing radiation laboratory, Radiation and Nuclear Safety Authority, Helsinki, Finland; ^e^ Unit of Primary Health Care, Helsinki University Central Hospital, Helsinki, Finland; ^f^ Department of General Practice, University of Helsinki, Helsinki, Finland; ^g^ Unit of Primary Health Care, Kuopio University Hospital, Helsinki and Kuopio, Finland; ^h^ Finnish Meteorological Institute, Arctic Research Centre, Sodankylä, Finland; ^i^ Aeronomy division, Sodankylä Geophysical Observatory, Sodankylä, Finland; ^j^ Children’s Hospital, Helsinki University Central Hospital and University of Helsinki, Helsinki, Finland

**Keywords:** 25(OH)D, vitamin D, ultraviolet, UVB, spring

## Abstract

Humans obtain vitamin D from conversion of 7-dehydrocholesterol in the skin by ultraviolet B (UVB) radiation or from dietary sources. As the radiation level is insufficient in winter, vitamin D deficiency is common at higher latitudes. We assessed whether vernal solar UVB radiation at latitudes 61°N and 67°N in Finland has an impact on serum 25-hydroxyvitamin D [S-25(OH)D] concentrations. Twenty-seven healthy volunteers participated in outdoor activities in snow-covered terrain for 4–10 days in March or April, with their face and hands sun-exposed. The personal UVB doses and S-25(OH)D levels were monitored. A mean UVB dose of 11.8 standard erythema doses (SED) was received during an average of 12.3 outdoor hours. The mean S-25(OH)D concentration in subjects with a baseline concentration below 90.0 nmol/L (n=13) increased significantly, by 6.0 nmol/L from an initial mean of 62.4 nmol/L (p<0.001), whereas in those with a basal concentration above 90.0 nmol/L (n=12) it decreased significantly, by 6.7 nmol/L from a mean of 116.9 nmol/L (p<0.01). To conclude, only 7% of total body surface area was exposed to vernal sunlight and this was capable of increasing S-25(OH)D levels in subjects with a baseline level below 90 nmol/L but not in those with higher levels.

## Introduction

Humans obtain vitamin D either from conversion of 7-dehydrocholesterol in the skin by ultraviolet B (UVB) radiation, or from dietary sources. Depending on the latitude, time of the year and the mode of subsistence, dietary sources can be the major form, especially for Northern peoples [1]. Vitamin D is hydroxylated in the liver to the circulating form, 25-hydroxyvitamin D [25(OH)D], which is the best indicator of vitamin D status. Vitamin D insufficiency has been linked to chronic skeletal [2] and extra-skeletal diseases such as obesity and type 2 diabetes mellitus [3,4]. Low S-25(OH)D levels are common in Scandinavians in winter, because the available sunlight is not capable of inducing vitamin D synthesis and their dietary intake of vitamin D is often suboptimal [5,6]. Although recent national policies have succeeded in increasing vitamin D intake in Finland, roughly a quarter of men and half of women fail to reach the recommended dietary intake [7]. It seems, however, that native populations that have lived at high latitudes for hundreds of generations have adapted to low S-25(OH)D levels by developing compensating mechanisms [1,8].

The lowest S-25(O)D levels in subjects living at high latitudes are typically measured between February and April [9–12]. In theory, there is enough sunlight for vitamin D synthesis from early March onwards at a latitude of 61°N [13]. A previous Norwegian study at latitude 68°N supported the theoretical calculations, since individual subjects with low S-25(OH)D responded to solar UV radiation already in early March when just the face was exposed, but the study cohort was very small [10]. Another Danish study at latitude 56°N showed an increase in S-25(OH)D by 8 April when more skin than the face and hands was exposed [11]. At higher latitudes, cold weather prevents people from exposing more skin than the face and hands during March and April, but exposure of these areas would seem to suffice to increase S-25(OH)D levels [10,14]. Reflections from snow-covered terrain can substantially increase the UVB dose received by the skin during outdoor activities [15], which can result in a higher UVB dose than measured previously [13]. Since previous studies are scarce, our goal was to determine whether the solar UVB radiation level at high latitudes in Finland in March and April is capable of raising S-25(OH)D concentrations when only the face and hands are exposed.

## Material and methods

### Subjects

Twenty-seven healthy volunteers were enrolled, the inclusion criteria being age 18 years or older and avoidance of solarium visits, phototherapy, holidays in low latitudes and vitamin D supplementation during a 1-month washout period prior to the trial and during it. Further exclusion criteria were pregnancy, previous skin cancer, intake of photosensitising drugs and Fitzpatrick´s skin phototype 1 [16]. Recruitment began on 1 February 2013 and the trial was coordinated from the Department of Dermatology at Päijät-Häme Central Hospital, terminating in data collection on 22 April 2014. Vitamin D intake at the onset was estimated by means of a 3-day food frequency questionnaire. Twenty-five subjects completed the trial (Table 1), two having been disqualified for failing to follow the exposure regimen. The protocol was approved by the Ethics Committee of Tampere University Hospital (Reg. No. R12266), and all the volunteers gave their informed consent in advance.

**Table 1.** Demographic data, daily vitamin D intake, baseline S-25(OH)D concentration and personal ultraviolet B radiation dose.

	N=25
Male/Female	8/17
Mean age, years (range)	43 [22–71]
Mean body mass index, kg m^−2^, mean±SD (range)	23.9±4.5
Fitzpatrick’s skin type II/III	7/18
Vitamin D intake, µg/d, mean±SD	8.5±3.2
Baseline S-25(OH)D (nmol/L), mean±SD	88.6±32.6
UV dosimeter (SED), mean±SD (range)	11.8±4.9 (2.4–23.2)

### Sample size calculation

The trial was designed to show an increase in S-25(OH)D of at least 15 nmol/L with an α-value of 0.05 and a β-value of 0.80. An assumed SD of 15.5 nmol/L for the S-25(OH)D analyses was used. Accordingly, it was considered necessary that 16 volunteers should complete the trial.

### Ultraviolet radiation exposures

The scheduled monitored ultraviolet radiation (UVR) exposures were implemented in Sodankylä (67°N) and Lahti (61°N) in March and April 2013 and 2014 in snow-covered terrain (Table 2). The participants were instructed to expose their hands and face without using a sunscreen. In addition to the scheduled exposures, they were encouraged to perform outdoor activities in their own time.

**Table 2.** Ultraviolet radiation exposure locations, time periods, exposure instructions and average daily available ultraviolet B radiation doses.

Group	Location	Dates	Daily available UVB radiation dose (SED), mean (range)	Maximum UV index	Personal UVB radiation dose (SED), mean ± SD N = 21	Total hours spent outdoors between 10AM and 3PM, mean ± SD
I (N=4)	Sodankylä, Finland (67°N, 26°E)	17.3.–26.3.2013^1^	5.0 (4.1–6.2)^3^	1.2	20.5^6^	19.3±3.0
II (N=6)	Sodankylä, Finland (67°N, 26°E)	29.3.–1.4.2013^1^	7.1 (6.8–7.3)^5^	1.2	11.1±7.6	12.2±3.8
III (N=5)	Sodankylä, Finland (67°N, 26°E)	7.4.–17.4.2014^2^	9.6 (4.5–13.2)^3^	2.3	10.9±1.7	11.0±3.2
IV (N=3)	Sodankylä, Finland (67°N, 26°E)	18.4.–20.4.2014^1^	13.0 (10.4–15.3)^3^	2.7	11.2±6.9	9.0±1.7
V (N=7)	Lahti, Finland (61°N, 25°E)	2.4.–12.4.2013^2^	12.1 (9.1–15.4)^4^	2.7	11.9^7^	10.7±2.5
Mean					11.8±4.9	12.3±4.3

^1^Maximum exposure during holiday outdoor activities. ^2^Walking outdoors for 1 hour daily at noon during working days. ^3^NILU-UV measurements from FMI-ARC, ^4^Spectroradiometer measurements from FMI, Jokioinen Observatory. ^5^Local Robertson-Berger meter. ^6^Only one subject had a dosimeter. ^7^One dosimeter for the whole group.

### Ultraviolet radiation measurements

The participants wore UVB dosimeters (VioSpor blue line Type II, BioSense, Bornheim, Germany) attached to their upper arms or wrists with straps to detect the dose received by the skin [17,18]. These dosimeters detect radiation ranging from 1.0 to 55 standard erythema doses (SED). The ambient solar UVR data were obtained from NILU-UV multichannel radiometer [19] recordings made at the Finnish Meteorological Institute’s Arctic Research Centre (FMI-ARC) in Sodankylä, or were measured locally using a Robertson-Berger-type broadband UV meter (Solar Light Model 501 UV meter s/n 635; Solar Light Co. Inc., Glenside, PA, USA). The NILU-UV radiometer was calibrated by Innovation Nilu AS by reference to the National Institute of Standards and Technology (NIST) (Gaithersburg, MD, USA) and was placed on the roof of the FMI-ARC sounding station to collect data in the form of 1-minute averages. The Robertson-Berger meter is calibrated annually by the Radiation and Nuclear Safety Authority, Helsinki, Finland, also by reference to NIST. Its calibration uncertainty (2σ) is 8%. The meter was placed on a high roof near the UV exposure area. As there were no ambient UVR measurements available for Lahti, use was made of UVR data obtained at Jokioinen (61°N) with the FMI’s Brewer spectroradiometer [20], which was calibrated by reference to the MIKES-Aalto National Standards Laboratory. Comparisons with the European reference spectroradiometer have shown that discrepancies are less than ±5% (http://www.pmodwrc.ch/wcc_uv/).

### Serum 25-hydroxyvitamin D measurements

The blood samples for 25(OH)D analyses were taken in general in the morning on the first exposure day (range 0–5 days before), and 1–4 days after the last day. The samples were centrifuged and serum was stored at –20°C and analysed for 25(OH)D by electro-chemiluminescence binding assay (Roche Diagnostics, Mannheim, Germany) with a coefficient of variation ≤7%.

### Statistics

The strength of the adjusted relationship between baseline S-25(OH)D concentrations and their change after the solar UVR exposure period was described by means of a partial correlation coefficient. The significance of the change was calculated using the paired-samples t-test, and Pearson’s chi-square test was used when comparing nominal data. All the analyses were performed using STATA 14 (StataCorp LP, College Station, TX).

## Results

**Figure 1.**
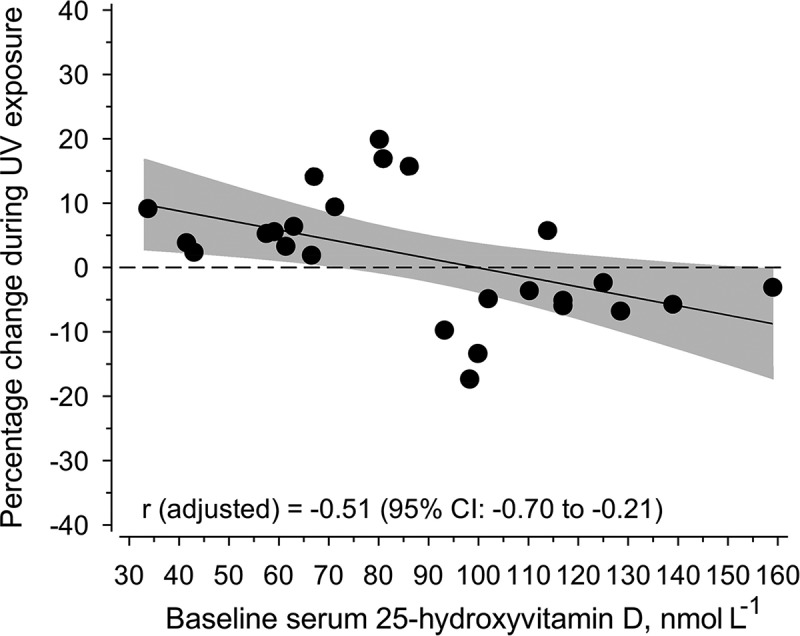
Relationship of changes in S-25(OH)D concentrations after the solar ultraviolet radiation exposure period to baseline concentrations. R adjusted for age, body mass index and Fitzpatrick’s skin type, p=0.011.

The mean daily vitamin D intake of the 25 subjects was 8.5±3.2 µg (Table 1) and the mean baseline S-25(OH)D concentration was 88.6±32.6 nmol/L. The mean personal total UVB dose was 11.8±4.9 SED during a mean of 12.3±4.3 hours spent outdoors (Tables 1 and 2). The mean available ambient daily UVB dose was 9.4 SED and the maximum UV indices were 1.1–2.7 depending on the period. The mean S-25(OH)D concentration showed a slight non-significant decrease of 0.1 nmol/L (p=0.971) after the exposures, but the baseline concentrations had an inverse relationship with the percentage change after exposure when adjusted for age, body mass index and Fitzpatrick’s skin phototype (r=−0.51, p=0.011) (Figure 1).

**Figure 2.**
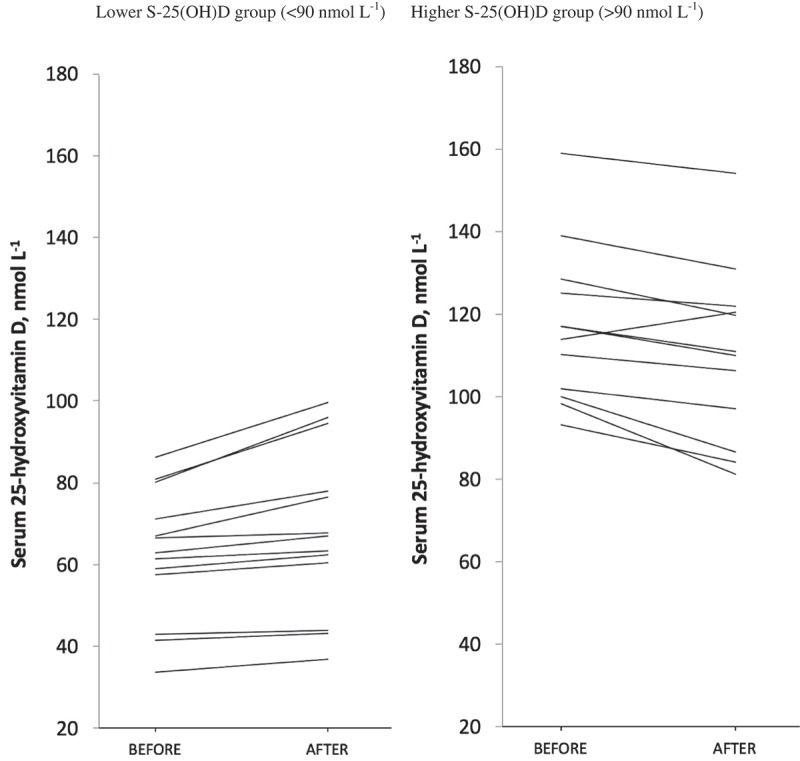
Changes in S-25(OH)D concentrations in the lower S-25(OH)D group (<90 nmol/L) and in the higher S-25(OH)D group (>90 nmol/L).

The baseline S-25(OH)D concentrations below 90.0 nmol/L increased after exposure, whereas those that were above 90.0 nmol/L decreased, in all subjects except one (Figure 2, Table 3). In the <90 nmol/L group (n=13) the S-25(OH)D increased by 6.0 nmol/L (95% CI 2.8–9.2, p<0.001) from an initial mean of 62.4 nmol/L and that in the >90 nmol/L group (n=12) decreased by 6.7 nmol/L (95% CI −10.3 to −3.0, p<0.01) from 116.9 nmol/L (Figure 2, Table 3). The total UVB doses received by the volunteers correlated with the numbers of hours spent outdoors (r=0.612, p=0.02), but no correlation was found between the UVB doses and the change in S-25(OH)D concentrations. There were no differences in demographic data or UVB doses detected between the subjects with a S-25(OH)D value below or above 90 nmol/L (Table 3). Four subjects with baseline S-25(OH)D below 90 nmol/L and six subjects with baseline S-25(OH)D above 90 nmol/L had used vitamin D supplementation prior the 1-month washout period with a mean daily dose of 13.3±7.3 µg (range 5–25 µg). The mean dose did not differ significantly between the groups (p=0.081). The baseline S-25(OH)D levels in males (83.3 nmol/L) and females (91.0 nmol/L) did not show a significant difference (p=0.593).

**Table 3.** Demographic data and changes in S-25(OH)D concentration after solar exposure in subjects with low (<90.0 nmol/L) and high (>90.0 nmol/L) baseline S-25(OH)D concentrations.

	Low S-25(OH)D (N=13)	High S-25(OH)D (N=12)	p-value
Male/Female	5/8	3/9	0.637
Mean age, years (range)	43 [22–63]	44 [24–71]	0.888
Mean body mass index, kg m^−2^, mean±SD (range)	25.0±5.2	22.3±3.1	0.204
Fitzpatrick’s skin type II/III	2/11	5/7	0.202
Vitamin D intake, µg/d, mean±SD	7.6±1.8	9.4±4.1	0.182
Baseline S-25(OH)D (nmol/L), mean±SD	62.4±15.8*	116.9±18.9**	
Change of the S-25(OH)D (nmol/L), mean (95% CI)	6.0 (2.8 to 9.2)*	−6.7 (−10.3 to −3.0)**	
S-25(OH)D after the exposure (nmol/L), mean±SD	68.4±20.3*	110.3±21.2**	
UV dosimeter (SED), mean±SD	11.4±5.0	12.4±5.0	0.642
Hours spent outdoors between 10 am and 3 pm, mean±SD	11.4±3.3	13.2±5.1	0.303

*Significant increase (p<0.001) **Significant decrease (p<0.01).

## Discussion

Our results indicate that vernal UVB radiation at a high latitude (61° or 67°) between 17 March and 20 April is capable of raising S-25(OH)D levels in subjects with baseline levels below 90 nmol/L, whereas levels above that decrease. The mean baseline level 88.6 nmol/L was higher than expected at high latitudes in early spring. This could be attributed to national food fortification policy and improved dietary vitamin D intake [7], use of vitamin D supplementation or holidays in low latitudes prior the washout period. In our previous study [21], where S-25(OH)D levels were monitored from October to May in subjects with no vitamin D supplementation during the winter, nor any exposure to UVR, the mean concentration in early April was lower, 65.8 nmol/L.

The increase of lower S-25(OH)D levels early in spring is a solid finding, but the decreasing trend in higher levels is unexpected, since all subjects were exposed to a source of vitamin D. It could be that in subjects with higher levels, the S-25(OH)D gained from previous vitamin D sources keeps decreasing due to the half-life of S-25(OH)D, and this decrease exceeds the amount of S-25(OH)D produced by vernal UVB. Interestingly, we further analysed data from our previous study [21] and observed similar kinetics in the control subjects – higher values kept on decreasing from early April to early May, whereas lower values began to increase. In fact, this phenomenon could also be due to an effective homeostatic S-25(OH)D control system for ensuring its stable availability. The points at which this regulation takes place evidently include (i) the liver concentration of 25-hydroxylase, which converts vitamin D to 25(OH)D, and (ii) catabolism of 25(OH)D to breakdown products in the liver and in other tissues [22]. The inverse relationship between the change in S-25(OH)D concentration and its baseline concentration in our subjects might in part be caused by this regulation. A similar phenomenon has been demonstrated also in other high latitude studies in the Nordic countries [10,12,23].

The homeostatic control system exists because of the health risks of having a S-25(OH)D concentration too low or high. It is generally agreed upon that a U-shaped response curve exists between the S-25(OH)D concentration and various disease risks. In a Swedish study, an approximately 50% higher total mortality rate was observed among men in the lowest 10% (≤46 nmol/L) and the highest 5% (≥98 nmol/L) of plasma 25(OH)D concentrations compared with intermediate concentrations [24]. S-25(OH)D below 40 nmol/L and above 60 nmol/L have shown to increase the risk of prostate cancer in the Finnish population, and both high and low levels seem to promote premature ageing [25]. A transnational study in women reported increased mortality for seven types of cancer (endometrial, oesophageal, gastric, kidney, non-Hodgkin’s lymphoma, pancreatic, ovarian) in subjects with S-25(OH)D below 45 nmol/L and above 124 nmol/L [26]. A pooled meta-analysis including eight case-control studies reported an increased pancreatic cancer risk in subjects with S-25(OH)D above 100 nmol/L [27]. The Framingham Heart Study concluded that cardiovascular disease risk increases with S-25(OH)D below 50 nmol/L and above 62.5 nmol/L, and the NHANES III study found a higher all-cause mortality rate for S-25(OH)D above 122.5 nmol/L [28].

Like most heritable characteristics, vitamin D metabolism varies among human populations. Populations that have lived at high latitudes for hundreds of generations have had time to adapt to limited opportunities for vitamin D synthesis. This kind of adaptation has been shown in the Inuit of Northern Canada and Greenland. Despite low S-25(OH)D levels and a calcium-deficient diet, Inuit have normal blood levels of calcium. They seem to absorb calcium more effectively, perhaps due to their different vitamin D receptor genotype prevalence [8]. They also seem to convert vitamin D at a higher rate to its most active form [1]. These metabolic differences may explain why Amerindian women have lower S-25(OH)D levels than do Euro-American women, while having higher bone mass density until menopause [29,30].

There is some worrisome evidence that native Northern people can experience effects of vitamin D toxicity at relatively low levels. A recent study of Greenland Inuit found increasing S-25(OH)D levels to be positively associated with increased fasting- and 2-hour plasma glucose and HbA1c, and decreased beta-cell function [31]. This dose-response curve was very different from the one seen in European populations. Although a homeostatic mechanism seems to keep S-25(OH)D levels within a zone of minimal health risks [22], this homeostasis could be circumvented through daily ingestion of high vitamin D supplementation doses. This is a relatively novel situation for our species, and the risks could be higher for those who have adapted to low S-25(OH)D levels.

As cutaneous vitamin D synthesis depends on the UVB dose received in the skin, factors that affect UVB wavelength and irradiance have a direct influence on vitamin D synthesis. The most relevant factor for Northern inhabitants is the solar zenith angle, which depends on the time of day, season of the year and latitude. As the zenith angle increases, the UVB radiation has to travel a longer distance through the atmosphere and has more chance of being absorbed or scattered, which reduces the amount reaching the skin [32]. At latitude 52°N no cutaneous vitamin D synthesis is detectable from October to March, a phenomenon described as the “vitamin D winter” [32]. The length of the “winter” is not constant, however, but varies with levels of ozone, cloudiness and aerosols [33]. Ozone levels can reduce or increase the latitude of the “vitamin D winter” by 10°, and extend or shorten its duration by up to 2 months [33]. The lowest seasonal S-25(OH)D concentrations in subjects living at high latitudes are typically measured between February and April [9–12]. In theory, cutaneous vitamin D production is possible at latitude 61°N from early March [13], an estimate based on UV spectroradiometer irradiance measurements on a horizontal surface, but a snowy surface can further increase the subject’s UVB dose due to reflections between the ground and sky and direct reflections from the ground to vertical surfaces [14]. The albedo of snow varies between 0.5 and 0.7 [34,35] and the effect is most pronounced for vertical surfaces such as the face [15]. All the outdoor activities performed by the present subjects took place in snow-covered fields, which probably increased the measured personal UVB doses significantly.

The UV exposures were received on the face and hands, the exposure of which has been shown to suffice to increase S-25(OH)D levels [10,14]. A previous study performed in Denmark (56°N) showed an increase in S-25(OH)D levels by 8 April, when more skin than the face and hands was exposed [11]. In a Norwegian study (68°N) exposure of the facial area to UVR between 8 February and 12 April yielded no increase in mean S-25(OH)D levels [9], being in agreement with our findings. Interestingly, subjects with S-25(OH)D <30 nmol/L responded in early March [10], whereas in our study the cut-off limit was 90 nmol/L. Our cut-off limit was higher probably because of a larger exposed skin area (4% vs. 7%), longer minimum daily exposure time (20 min vs. 60 min) and study period that extended further towards the spring (20 April). Contrary to the previous study [10], in our subjects the increase of S-25(OH)D levels below 90 nmol/L was highly significant and strengthens the evidence of the vitamin D effects of vernal UVR. However, the optimal S-25(OH)D level can differ between populations, and thus the findings of the present study are not directly generalisable.

To conclude, vernal solar UVR at high latitudes seems to increase S-25(OH)D concentrations in subjects with a baseline level below 90 nmol/L when only the face and hands, i.e. 7% of total body surface area, are exposed. The declining trend seen in volunteers with a high baseline level above 90 nmol/L is interesting, and could reflect an especially active homeostatic S-25(OH)D control system in caucasoid people living at high latitudes. This phenomenon should be studied in more detail in future trials.
